# Monthly Summary

**Published:** 1889-12

**Authors:** 


### Monthly Summary.

---

GONORRHŒA AT FIVE YEARS OF AGE.—DEATH AFTER NITROUS OXIDE ANÆSTHESIA.—REUNION OF CUT-OFF FINGERS.—In the REPORTER of October 19, a case of gonorrhœa in a boy five years old is reported by Dr. D. D. Custer, of Manayunk, Pa. In 1877 a woman brought her son, aged five years, to me, saying that something was wrong with him; that he cried when he urinated, and this was quite frequent. On examination I found he had a well-defined case of gonorrhœa. Investigation proved that the hired girl had seduced him, and he contracted gonorrhœa from her. My partner, at the time, was treating a young man who contracted the disease from the same woman.

In the REPORTER of November 2, you mention a death from nitrous oxide gas, or rather from apoplexy following administration of the gas. Several years ago I administered nitrous oxide gas to a lady, for a dentist to extract some teeth, and she came near dying from what I then considered threatened apoplexy. The case you mentioned reminded me very forcibly of my experience then, and I hope never to have a repetition of it. In the same issue of the REPORTER, Dr. A. Hamilton Deekens reports the union of a cut-off finger, which reminds me of what happened to an old physician many years ago; long before antiseptic surgery was practiced. A man, with a finger cut off, came to him, bringing the finger. The doctor was drunk, and sewed the finger back. It united nicely. But lo! the doctor had sewed it on with the palm surface turned the wrong way. The doctor, after sobering up, wanted to amputate the finger and try to put it back right, but the patient declined and the doctor was annoyed, many years, by having his mistake constantly exhibited as a great curiosity.

Yours truly,

S. W. SANFORD, M. D.

Henning, Tenn.—*Medical and Surgical Reporter.*

---

DEATH FROM CHLOROFORM.—A death from the inhalation of chloroform occurred at the Richmond Hospital, London, Oct. 12. The patient was a woman, who was about to undergo amputation of the thumb. She had taken a very ew inspirations when her face was observed to become deeply congested. The administration of the anæsthetic was at once stopped, artificial respiration was set up, and the external jugular vein was opened, but she never rallied. At the autopsy examination the heart was found to be infiltrated with fat and the brain to be congested. The coroner's jury found that the anæsthetic was skillfully administered.—*British Medical Journal,* Oct. 12, 1889.

